# Detection of Batborne Hantaviruses, Laos, 2023–2024

**DOI:** 10.3201/eid3104.241720

**Published:** 2025-04

**Authors:** Chittaphone Vanhnollat, Somphavanh Somlor, Kristina Dimitrova, Sarah Medina, Khamsing Vongphayloth, Vaekey Vungkyly, Longthor Vachouaxiong, Bounsavane Douangboubpha, Daosavanh Sanamxay, Vilakhan Xayaphet, Phetphoumin Paphaphanh, Watthana Theppangna, Jonathan Audet, Philippe Buchy, David Safronetz

**Affiliations:** Institut Pasteur du Lao People’s Democratic Republic, Vientiane, Laos (C. Vanhnollat, S. Somlor, K. Vongphayloth, V. Vungkyly, L. Vachouaxiong, P. Buchy); National Microbiology Laboratory, Public Health Agency of Canada, Winnipeg, Manitoba, Canada (K. Dimitrova, S. Medina, J. Audet, D. Safronetz); National University of Lao People’s Democratic Republic, Vientiane (B. Douangboubpha, D. Sanamxay, V. Xayaphet, P. Paphaphanh); National Animal Health Laboratory Center, Ministry of Agriculture and Forestry, Vientiane (W. Theppangna)

**Keywords:** hantavirus, viruses, batborne virus, zoonoses, vector-borne infections, ecology, Chiroptera, Laos

## Abstract

We report the detection of batborne hantaviruses in 2 bat species (*Aselliscus stoliczkanus* and *Hipposideros gentilis*) in Laos, expanding the known geographic distribution of hantaviruses in Southeast Asia. Given the frequent human–wildlife contact in the region, researchers should continue to characterize the viruses and investigate their zoonotic potential.

The Hantaviridae family currently includes 8 genera and 53 species (*1*). Many of those viruses, particularly those in the genus *Orthohantavirus*, are typically associated with rodent hosts and are known to cause severe diseases in humans, including hemorrhagic fever with renal syndrome and hantavirus pulmonary syndrome. However, since the late 2000s, hantaviruses have been discovered in various nonrodent species, including shrews (e.g., *Suncus murinus*, *Crocidura shantungensis*), moles (e.g., *Scalopus aquaticus*, *Talpa europaea*), and various bats (e.g., *Rousettus amplexicaudatus*, *Rhinolophus affinis*). Those discoveries have substantially expanded our knowledge of hantavirus ecology and evolutionary origins (*2*).

Laos is in a tropical region recognized as a hotspot for emerging and reemerging infectious diseases (*3*). The country faces several zoonotic risk factors, including a high rate of human–wildlife contact and illegal wildlife trade, particularly involving species at high risk for zoonoses (*4*). Serologic and molecular evidence has shown that rodentborne hantaviruses circulate in Laos (*5*), but batborne hantaviruses have not been reported, although researchers have identified several batborne hantaviruses in neighboring countries, including China (Laibin, Huangpi, and Longquan viruses), Myanmar (Laibin virus), Vietnam (Dakrong virus [DKGV] and Xuan Son virus [XSV]), and the Philippines (Quezon virus) (*6*). Those batborne hantaviruses are assigned to the *Loanvirus* and *Mobatvirus* genera of the Hantaviridae family (*7*). In this study, we explored the possible presence of batborne hantaviruses in Laos.

## The Study

**Figure 1:**
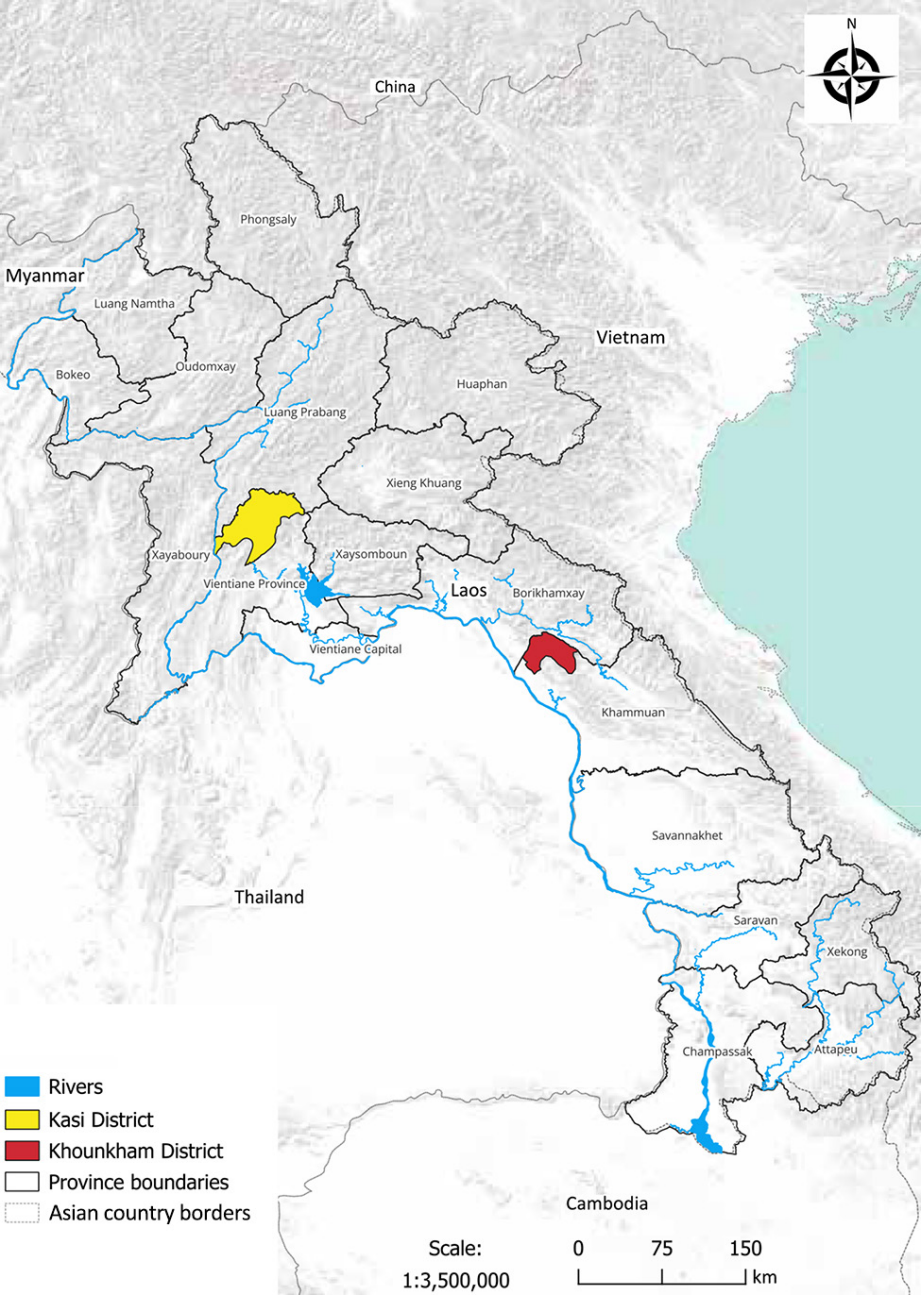
Sampling locations in Kasi and Khounkham districts for study of detection of batborne hantaviruses, Laos, 2023–2024.

**Figure 2:**
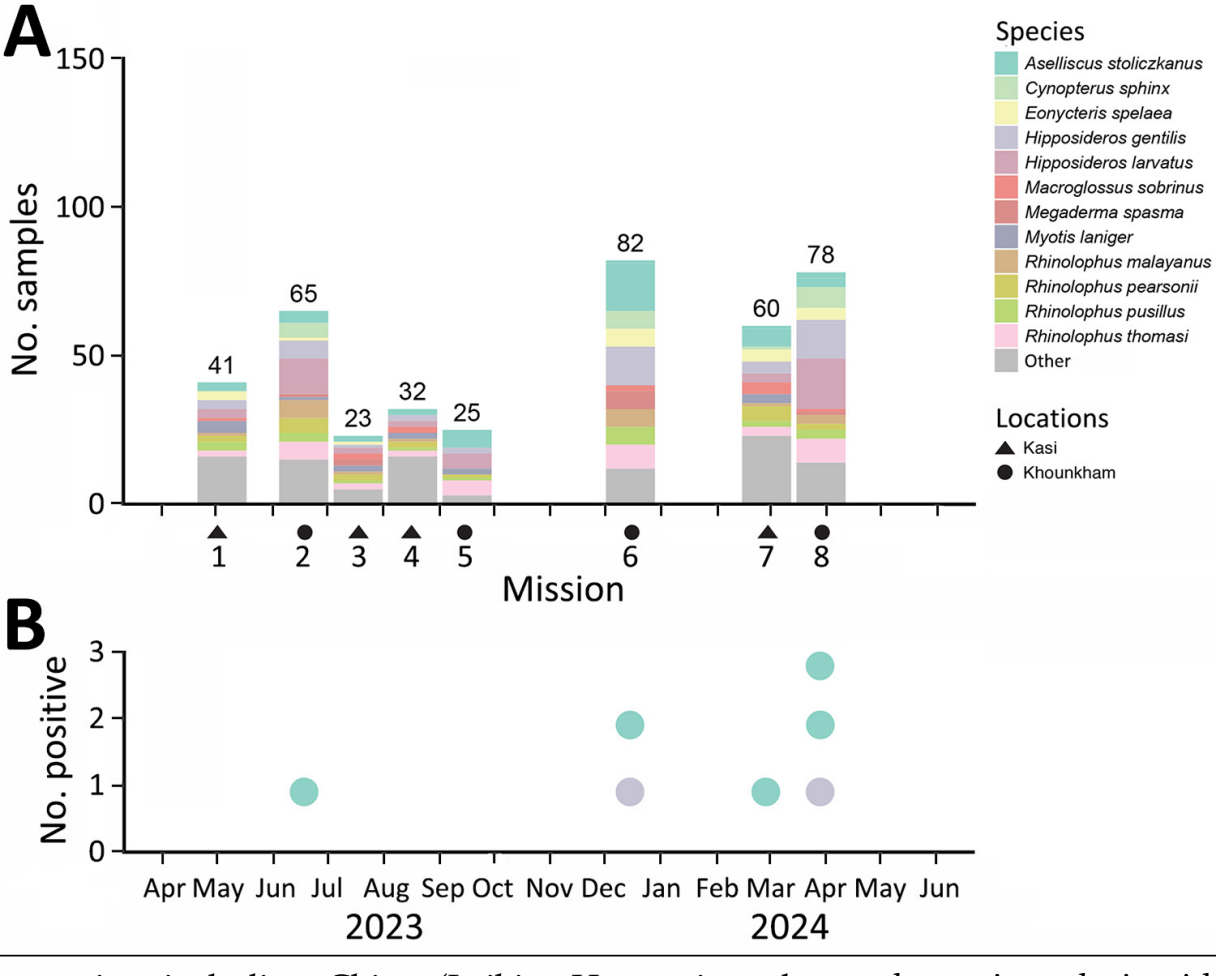
Sampling of bat species for study of detection of batborne hantaviruses, Laos, 2023–2024. A) Number of samples collected by species and location. Other represents bat species for which <10 bats were captured during each field mission. B) Number of positive samples collected during the sample period.

We conducted this research with the approval of the animal health authorities of the Department of Livestock and Fisheries, Ministry of Agriculture and Forestry (Vientiane, Laos) (approval no. 0981/DLF, issued on April 21, 2023). As part of a field ecologic study, we carried out 8 missions longitudinally at 2 locations during May 2023–April 2024: Khounkham district in Khammouane Province (18.16N, 104.47E) and Kasi district in Vientiane Province (19.13N, 102.12E) (Figures 1, 2). We selected those sites to represent 2 distinct landscapes of limestone karsts, characterized by the presence of caves, sinkholes, and underground drainage systems. Khounkham is an interconnected area, and Kasi is an isolated area. 

We captured 2,814 bats by using 4-bank harp traps and mist nets, targeting known or suspected flight paths. We initially identified bat species on the basis of key morphologic characteristics at the time of collection, as previously described (*8*). We later used genetic analysis with mitochondrial cytochrome B to confirm species for specific bats (*9*). All personnel involved in bat handling and sample collection wore eye protection, nonvalved N95 respirators, fluid-resistant protective clothing, and double gloves. We followed the Canadian Council on Animal Care guidelines (https://ccac.ca/en/guidelines-and-policies/the-guidelines) for sampling and euthanizing captured animals. 

Of the 2,814 bats captured, we selected a subset of 406 bats for tissue collection on the basis of taxonomic and ecologic information. Captured bats represented 40 species and 18 genera and accounted for approximately half the bat species documented in Laos (*10*) (Figure 2; Appendix Table 1). To detect hantaviruses, we used total nucleic acid extraction, reverse transcription (*8*), and then a nested PCR (*11*) to test tissue samples (consisting of pooled heart, lung, liver, spleen, and kidney tissues) from each of the 406 bats. We used oligonucleotide primers, including both previously published and custom-designed primers, to obtain sequences of the small, medium, and large gene (L) segments (Appendix Table 2).

Overall, we detected hantaviruses, tentatively designated as Lao batborne hantaviruses 1–7 (LBHV-1–7), in 7 bats. Infected bats belonged to 2 species. Five positive samples (LBHV-1, -2, and -4–6) were from the Stolizka’s trident bat (*Aselliscus stoliczkanus*), and 2 (LBHV-3 and -7) were from the Andersen’s leaf-nosed bat (*Hipposideros gentilis*, previously known as *Hipposideros pomona*) (Appendix Figure 1). We trapped 1 infected bat in the Kasi district and the other 6 in the Khounkham district (Figure 2; Appendix Table 1).

**Figure 3:**
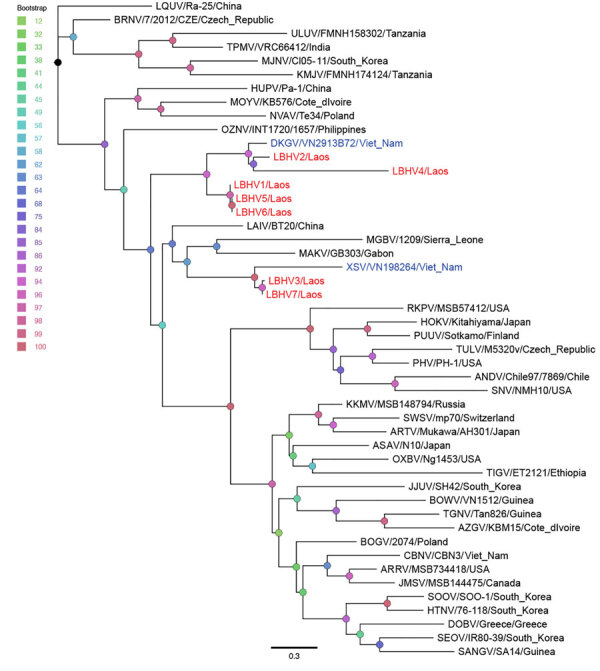
Phylogenetic analysis of batborne hantaviruses identified in Laos, 2023–2024, and reference sequences. Maximum-likelihood tree based on 300-bp partial RNA-dependent RNA polymerase sequence of the large (L) segment from LBHV-1–7 (red text) and other hantaviruses. Blue text indicates hantavirus strains from Vietnam. Sequences were aligned by using MAFFT version 7.520 (https://mafft.cbrc.jp/alignment/software) in auto mode. The tree was reconstructed with IQ-TREE version 2.3.2 (http://www.iqtree.org) by using the general time-reversible plus empirical base frequency plus proportion of invariable sites plus discrete Gamma model with default 4 rate categories substitution model, with 1,000 bootstrap replicates. Scale bar indicates nucleotide substitutions per site. LBHV, Lao batborne hantavirus. Additional virus abbreviations are given in the Appendix.

**Figure 4:**
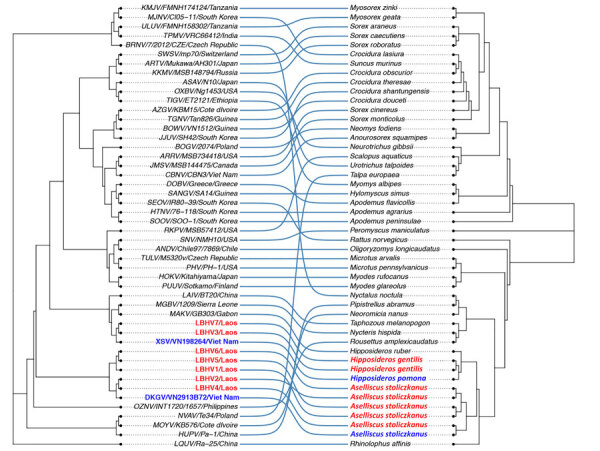
LBHVs and other mobatviruses from Southeast Asia showing detection of batborne hantaviruses, Laos, 2023–2024. The tanglegram compares maximum-likelihood trees from the hantavirus phylogeny in Figure 3 and the host cytochrome B gene. Red text indicates hantaviruses detected in Laos, and blue text indicate strains from Vietnam. The maximum-likelihood tree of the host cytochrome B gene was reconstructed with the same method as in Figure 3 with the AC = AT, CG = GT and equal base frequency plus proportion of invariable sites plus discrete Gamma model with default 4 rate categories model for cytochrome B, with 1,000 bootstrap replicates. LBHV, Lao batborne hantavirus. Additional virus abbreviations are given in the Appendix.

Phylogenetic analysis of the 300-bp amplicon sequences of the partial RNA-dependent RNA polymerase gene of the L segment showed that the newly detected hantaviruses from Laos clustered with other mobatviruses previously identified in Vietnam. Specifically, LBHV-1, -2, and -4–6 seemed to be phylogenetically related to DKGV, whereas LBHV-3 and -7 grouped with XSV (Figure 3). We similarly obtained partial coding sequences of the small and medium segments for LBHV-1 and -5–7. Phylogenetic analysis of those fragments was consistent with the L segment results, showing that LBHV-1, LBHV-5, and LBHV-6 grouped with DKGV, whereas LBHV-7 clustered with XSV (Appendix Figures 2, 3). Of note, those LBHV strains shared the same host species as the species from Vietnam within their respective clades. We detected LBHV-1, LBHV-2, and LBHV-4–6 in *A. stoliczkanus* bats, the same species hosting DKGV in Vietnam (*12*), and LBHV-3 and LBHV-7 in *H. gentilis* bats, the same species hosting XSV (*13*). We observed that pattern longitudinally across multiple field ecologic missions (Figure 2). In addition, co-phylogeny mapping on the basis of partial RNA-dependent RNA polymerase and bat cytochrome B nucleotide sequences showed that the phylogenetic groupings of LBHVs, DKGV, and XSV were highly congruent with their respective host species, supporting the pattern of host specificity and a close evolutionary relationship among those mobatviruses and their hosts (Figure 4, Appendix). That observation aligns with the established knowledge of host–hantavirus evolutionary relationships, where highly similar hantaviruses are typically found in similar host species rather than in more divergent hosts (*14*). However, further sequence information is still needed for a more detailed molecular and evolutionary characterization of the detected LBHVs. That information will help identify potential recombination, reassortment events, and genetic variations in other genome segments that might influence virus–host interactions and antigenic properties.

## Conclusions

This longitudinal study documented batborne hantaviruses in Laos, expanding the known geographic range of those viruses in Southeast Asia. Our findings show that the detected LBHVs are phylogenetically related to previously identified mobatviruses from Vietnam, specifically DKGV and XSV. Co-phylogeny mapping showed a clear host specificity, and each genetically related group associated with the same host species, suggesting a host–pathogen relationship. Given the geographic distribution of *A. stoliczkanus* and *H. gentilis* bats, which spans Myanmar, southern China, Laos, Vietnam, Cambodia, and western Malaysia (*10*), detection of similar isolates among bat species–associated mobatviruses in multiple locations is not surprising. However, further studies on virus identity and home range and migration patterns of their hosts are needed to determine whether the geographic distribution of those host-specific and genetically related viruses is caused by co-divergence or transmission among bats in different locations.

To clarify the underlying mechanisms of intraspecies viral maintenance and transmission, future investigations are needed to confirm whether bats carrying LBHVs are long-term residents or merely passing through the area where they were detected. Because hantaviruses are notoriously difficult to propagate in cell lines, and the ratio of viral to host genome is low in tissue samples from healthy bats, obtaining full-genome sequences is challenging. Although the zoonotic potential of LBHV remains unknown, the high rate of human–wildlife contact in the region highlights the need for future research. The public health focus should be on obtaining more sequence data and investigating the host and environmental factors that may contribute to virus persistence and spillover potential. Given the frequent human–wildlife contact in the region, researchers should continue to characterize the viruses and investigate the zoonotic potential of LBHV in Laos. Gaining insights into the ecology and transmission dynamics of these newly identified batborne hantaviruses could enhance our ability to rapidly diagnose and respond to future outbreaks caused by emerging hantaviruses. 

Appendix Taxa, sampling, and primer details for detection of batborne hantaviruses, Laos, 2023–2024.
