# Supplementary material for: Detection of Batborne Hantaviruses, Laos, 2023–2024
Source: Emerg Infect Dis. 2025 Apr;31(4):814–9. doi: 10.3201/eid3104.241720 (PMC11950268; doi:10.3201/eid3104.241720)
Supplement: Appendix — Taxa, sampling, and primer details for detection of batborne hantaviruses, Laos, 2023–2024. [file 24-1720-Techapp-s1.pdf]

Article DOI: https://doi.org/10.3201/eid3104.241720

EID cannot ensure accessibility for supplementary materials supplied by authors. Readers who have difficulty accessing supplementary content should contact the authors for assistance.

Detection of Batborne Hantaviruses, Laos, 2023–2024

Appendix

Taxa Information

Batborne hantaviruses 1–7 from Laos (large segment [L]: PQ585418–24, medium segment [M]: [PV034707-10], small segment [S]: [PV034711-14]). Other viruses: Longquan virus (LQUV Ra-25, L: JX465381, M: JX465397, S: JX465415), Huangpi virus (HUPV Pa-1, L: JX465369, S: JX473273), Mouyassue virus (MOYV KB576, L: JQ287716), Nova virus (NVAV Te34, L: KR072623, M: KR072622, S: KR072621), Ash River virus (ARRV MSB734418, L: EF619961, S: EF650086), Jemez Springs virus (JMSV MSB144475, L: FJ593501, M: FJ593500, S: FJ593499), Cao Bang virus (CBNV CBN3, L: EF543525.2, M: EF543526, S: EF543524), Seoul virus (SEOV IR80–39, L: NC_5238, M: NC_005237, S: NC_005236), Sangassou virus (SANGV SA14, L: JQ082302, M: JQ082301, S: JQ082300), Dobrava virus (DOBV Greece, L: NC_5235, M: NC_005234, S: NC_005233), Soochong virus-1 (SOOV SOO-1, L: DQ056292, M: AY675353, S: AY675349), Hantaan virus (HTNV 76–118, L: NC_5222, M: NC_005219, S: NC_005218), Boginia virus (BOGV 2074, L: JX990965, M: JX990966), Tanganya virus (TGNV Tan826, L: EF050454, M: EF050453, S: EF050455), Azagny virus (AZGV KBM15, L: JF276228, M: JF276227, S: JF276226), Bowe virus (BOWV VN1512, L: KC631784, M: KC631783, S: KC631782), Jeju virus (JJUV SH42, L: HQ663935, M: HQ663934, S: HQ663933), Seewis virus (SWSV mp70, L: EF636026, M: EF636025, S: EF636024), Amga virus (ARTV Mukawa, L: KF974361, M: KF974359, S: KF974360), Kenkeme virus (KKMV MSB148794, L: GQ306150, M: GQ306149, S: GQ306148), Oxbow virus (OXBV Ng1453, L: FJ593497, M: FJ539167, S: FJ539166), Tigray virus (TIGV ET2121, L: KU934008, M: KU934009, S: KU934010), Asama virus (ASAV N10, L: EU929078, M: EU929075, S: EU929072), Tula virus (TULV M5320v, L: NC_5226, M: NC_005228, S: NC_005227), Prospect Hill virus (PHV PH-1, L: EF646763, M: X55129, S: Z49098), Andes virus (ANDV Chile97, L: AF291704.5, M:

NC_003467, S: NC_003466), Sin Nombre virus (SNV NMH10, L: NC_5217, M: NC_005215, S: NC_005216), Hokkaido virus (HOKV Kitahiyama, L: AB712372, M: AB676848, S: AB675463), Puumala virus (PUUV Sotkamo, L: NC_5225, M: NC_005223, S: NC_005224), Rockport virus (RKPV MSB57412, L: HM015221, M: HM015219, S: HM015223), Hantavirus MGB/1209 (MGBV 1209, L: JN037851), Makokou virus (MAKV GB303, L: KT316176), Xuan son virus (XSV VN198264, L: JX912953, M: KU976427, S: KC688335), Laibin virus (LAIV BT20, L: KM102249, M: KM102248, S: KM102247), Đakrông virus (DKGV VN2913B72, L: MG663536, M: MG663535, S: MG663534), Quezon virus (OZNV INT1720, L: KU950715, M: KU950714, S: KU950713), Brno virus (BRNV 7, L: KX845680, M: KX845679, S: KX845678), Uluguru virus (ULUV FMNH158302, L: JX193697, M: JX193696, S: JX193695), Thottapalayam virus (TPMV VRC66412, L: EU001330, M: EU001329, S: AY526097), Imjin virus (MJNV Cl05–11, L: EF641806, M: EF641798, S: EF641804), Kilimanjaro virus (KMJV FMNH174124, L: JX193700, M: JX193699, S: JX193698).

Animal species: *Aselliscus stoliczkanus* (KU161570), *Hipposideros pomona* (JX912954), *Hipposideros ruber* (EU934474), *Taphozous melanopogon* (LC406449), *Rousettus amplexicaudatus* (KU950716), *Rhinolophus affinis* (JX465358), *Nyctalus noctula* (JX570902), *Neoromicia nanus* (JQ287717), *Pipistrellus abramus* (JX465352), *Nycteris hispida* (HQ693722), *Myosorex geata* (JX193701), *Myosorex zinki* (JX193702), *Suncus murinus* (DQ630386), *Crocidura lasiura* (KJ004674), *Crocidura shantungensis* (HQ663932), *Crocidura obscurior* (JF276229), *Crocidura theresae* (DQ521043), *Crocidura douceti* (KC684929), *Anourosorex squamipe* (AB175090), *Sorex cecutiens* (KF974362), *Sorex roboratus* (AB175128), *Sorex cinereus* (FJ667512), *Sorex monticolus* (FJ667514), *Sorex araneus* (FJ667524), *Neomys fodiens* (KC537797), *Urotrichus talpoides* (EU918371), *Scalopus aquaticus* (HM461914), *Talpa europaea* (FJ715340), *Neurotrichus gibbsii* (FJ595237), *Apodemus agrarius* (AB303225), *Apodemus flavicollis* (JF819967), *Apodemus peninsulae* (AB073811), *Rattus norvegicus* (AB355903), *Hylomyscus simus* (DQ212188), *Stenocephalemys albipes* (AF518346), *Myodes glareolus* (JX477304), *Myodes rufocanus* (LC406450), *Microtus pennsylvanicus* (AF119279), *Microtus arvalis* (AY220770), *Oligoryzomys longicaudatus* (AF346566), *Peromyscus maniculatus* (AF119261). The sequences were derived from the National Center for Biotechnology Information GenBank database (https://www.ncbi.nlm.nih.gov/genbank/).

References

1. Arai S, Aoki K, Sơn NT, Tú VT, Kikuchi F, Kinoshita G, et al. Đakrông virus, a novel mobatvirus (*Hantaviridae*) harbored by the Stoliczka's Asian trident bat (*Aselliscus stoliczkanus*) in Vietnam. Sci Rep. 2019;9:10239. PubMed https://doi.org/10.1038/s41598-019-46697-5

2. Klempa B, Fichet-Calvet E, Lecompte E, Auste B, Aniskin V, Meisel H, et al. Hantavirus in African wood mouse, Guinea. Emerg Infect Dis. 2006;12:838–40. PubMed https://doi.org/10.3201/eid1205.051487

Appendix Table 1. Detailed sampling numbers*

Species	Mission 1		Mission 2			Mission 3		Mission 4		Mission 5		Mission 6			Mission 7			Mission 8			Total
	–	Total	–	+	Total	–	Total	–	Total	–	Total	–	+	Total	–	+	Total	–	+	Total	
Aselliscus stoliczkanus	3	3	3	1	4	2	2	2	2	6	6	16	1	17	6	1	7	3	2	5	46
Cynopterus bracyotis												1		1							1
Cynopterus horsfieldii												5		5				1		1	6
Cynopterus sphinx			5		5							6		6	1		1	7		7	19
Eonycteris spelaea	3	3	1		1	1	1					6		6	4		4	4		4	19
Hesperoptenus blanfordi															1		1				1
Hipposideros armiger	2	2	1		1			1	1			1		1				4		4	9
Hipposideros gentilis	3	3	6		6	1	1	2	2	2	2	12	1	13	4		4	12	1	13	44
Hipposideros khaokhouayensis	1	1						1	1						2		2				4
Hipposideros larvatus	3	3	12		12	2	2	2	2	5	5				3		3	17		17	44
Hipposideros scutinares	2	2																1		1	3
Kerivoula depressa								1	1												1
Lyroderma lyra			1		1																1
Macroglossus sobrinus	1	1				2	2	2	2			2		2	4		4	1		1	12
Megaderma spasma			1		1	2	2					6		6				1		1	10
Megaerops niphanae			4		4	1	1											1		1	6
Miniopterus magnater	1	1				2	2	1	1						2		2				6
Murina cyclotis	1							2	2												2
Murina eleryi								1	1												1
Murina feae								1	1						1		1				2
Myotis alticraniatus	1	1				2	2	2	2						4		4				9
Myotis annectans	1	1													3		3				4
Myotis laniger	4	4	1		1	2	2	2	2	2	2				3		3				14
Myotis muricola	1	2						1	1						1		1				4
Myotis sp.			2		2					3	3							4		4	9
Phoniscus jagorii																		1		1	1
Rhinolophus episcopus								1	1												1

Species	Mission 1		Mission 2			Mission 3		Mission 4		Mission 5		Mission 6			Mission 7			Mission 8			Total
	–	Total	–	+	Total	–	Total	–	Total	–	Total	–	+	Total	–	+	Total	–	+	Total	
Rhinolophus malayanus	1	1	6		6	1	1	1	1			6		6	1		1	3		3	19
Rhinolophus marshalli	1	1																			1
Rhinolophus microglobosus	2	2	2		2			1	1						2		2				7
Rhinolophus pearsonii	2	2	5		5	2	2	2	2	1	1				5		5	2		2	19
Rhinolophus perniger	2	2						1	1						2		2	1		1	6
Rhinolophus pusillus	3	3	3		3	1	1	1	1	1	1	6		6	2		2	3		3	20
Rhinolophus rex			3		3							3		3	1		1				7
Rhinolophus siamensis	1	1										2		2							3
Rhinolophus thomasi	2	2	6		6	2	2	2	2	5	5	8		8	3		3	8		8	36
Rousettus sp.			2		2													1		1	3
Sphaerias blanfordi															2		2				2
Tylonycteris fulvida								2	2						1		1				3
Tylonycteris tonkinensis															1		1				1
Total	41	41	64	1	65	23	23	32	32	25	25	80	2	82	59	1	60	75	3	78	406

*Empty cells represent no available samples. –, negative; +, positive.

Appendix Table 2. List of primers used for the amplification of partial small, medium, and large segments

Primer name	Sequences, 5′–3′	Strand	Reference
Small segment			
S_clust0_man0_L	AAAAGGAGGTRGCAYTRGCA	Forward	This study
S_clust0_man1_R	TCTGCATCCATATCRTCCCCAAGA	Reverse	This study
S_clust1_man2_L	ATTGAGGARCCWTCTGGTGCAT	Forward	This study
S_clust1_man1_R	CCCCTAGTAGTATGCTCCYTAGT	Reverse	This study
Medium segment			
M_clust0_man1_L	TSTGCCAAAGGATYGACACAGA	Forward	This study
M_clust0_man1_R	GCYGCCCARCAYAACATTTCTA	Reverse	This study
T-M1199F	TAAVTTCAMCAACATGTC	Forward	(*1*)
T-M1485R	CCAGCCAAARCARAATGT	Reverse	(*1*)
Large segment			
HAN-L-F1	ATGTAYGTBAGTGCWGATGC	Forward	(*2*)
HAN-L-R1	AACCADTCWGTYCCRTCATC	Reverse	(*2*)
HAN-L-F2	TGCWGATGCHACIAARTGGTC	Forward	(*2*)
HAN-L-R2	GCRTCRTCWGARTGRTGDGCAA	Reverse	(*2*)

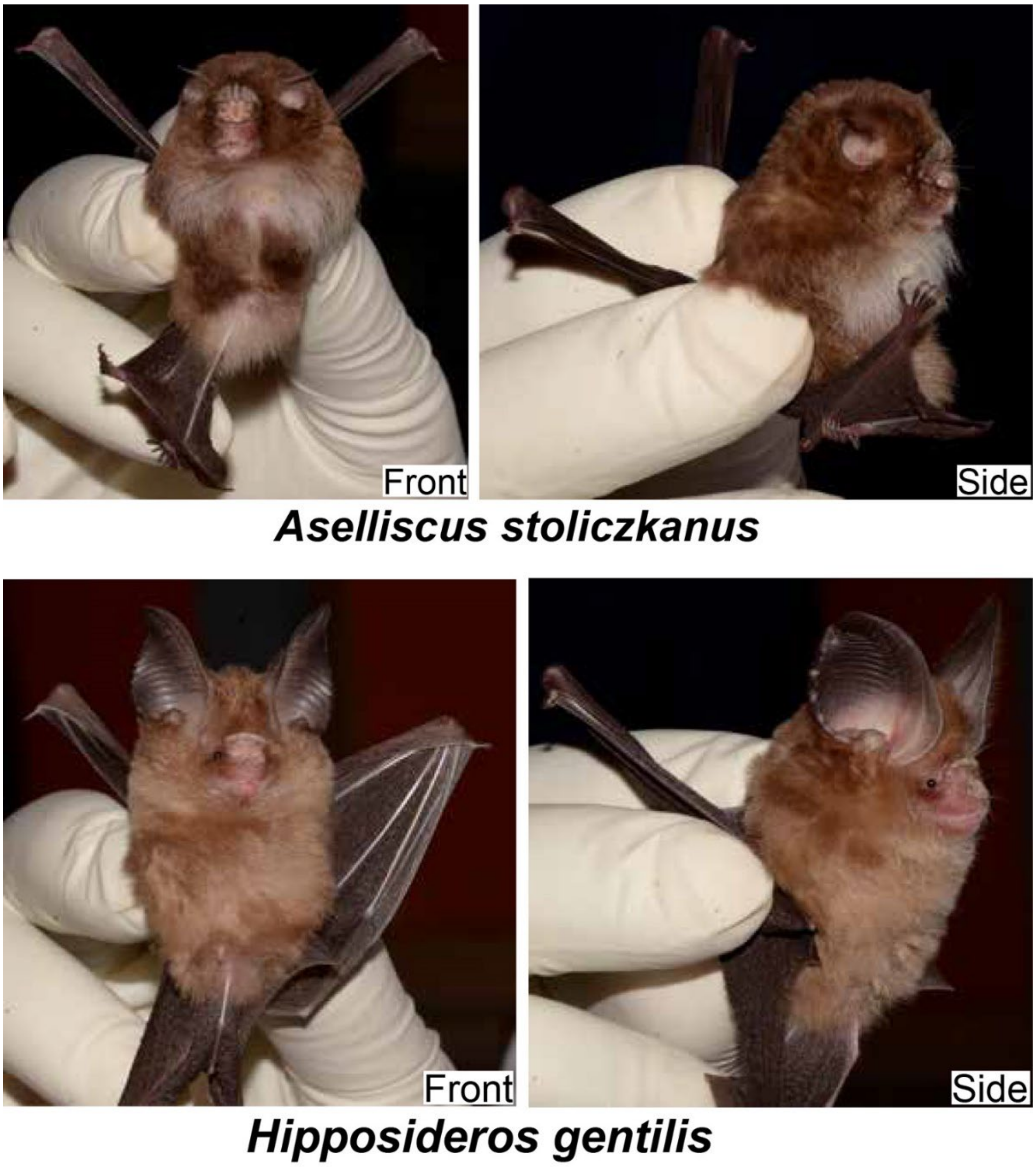

Appendix Figure 1. Photos of bat species that tested positive for batborne hantaviruses from Laos, 2023–2024.

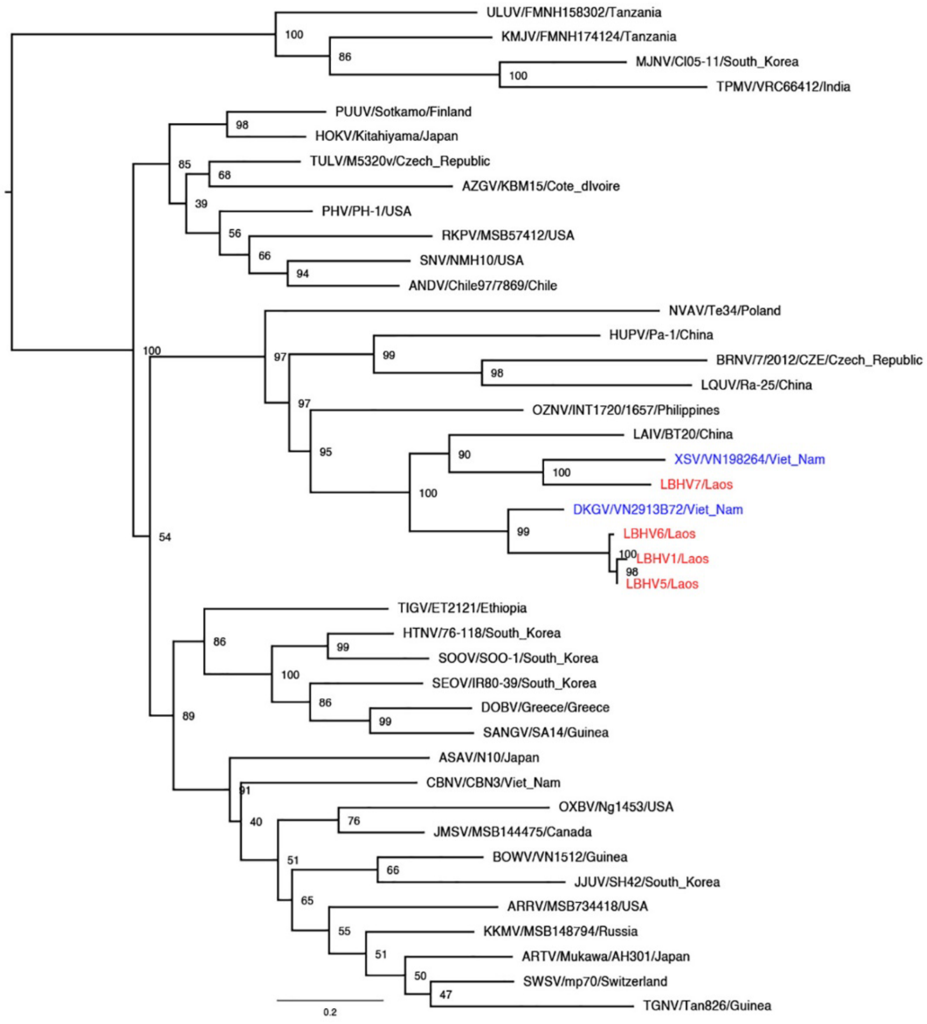

Appendix Figure 2. Phylogenetic tree of partial small (S) segments (≈200–700 bp) from LBHV-1 and -5–7 and other hantaviruses. Tree tips highlighted in red represent hantaviruses detected in Laos, and tips highlighted in blue indicate strains from Vietnam. The maximum-likelihood tree was reconstructed with the same method in Figure 3.

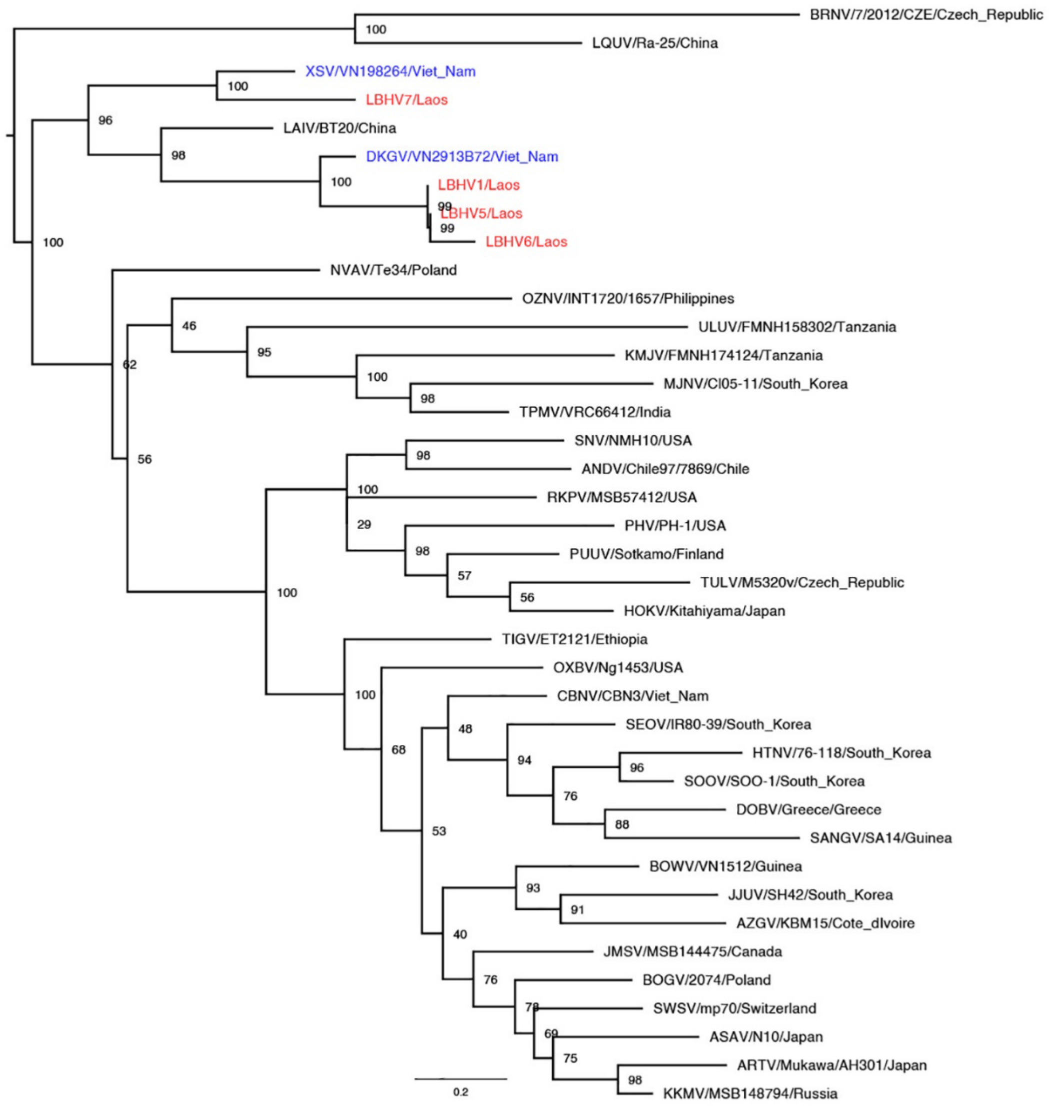

Appendix Figure 3. Phylogenetic tree of partial medium (M) segments (≈200–700 bp) from LBHV-1 and -5–7 and other hantaviruses. Tree tips highlighted in red represent hantaviruses detected in Laos, and tips highlighted in blue indicate strains from Vietnam. The maximum-likelihood tree was reconstructed with the same method in Figure 3.
